# Managing the unexpected: The role of homeless service providers during the 2017–2018 California wildfires

**DOI:** 10.1002/jcop.22653

**Published:** 2021-07-12

**Authors:** June L. Gin, Michelle D. Balut, Claudia Der‐Martirosian, Aram Dobalian

**Affiliations:** ^1^ U.S. Department of Veterans Affairs Veterans Emergency Management Evaluation Center (VEMEC) North Hills California USA; ^2^ Division of Health Systems Management and Policy, School of Public Health University of Memphis Memphis Tennessee USA

**Keywords:** community‐based organizations, disasters, emergency management, homelessness, housing, sheltering, wildfires

## Abstract

People experiencing homelessness during the 2017–2018 California wildfires faced significant risks of disruption. Homeless service organizations (HSOs) are an essential safety net for this population. To learn about how HSOs performed during the wildfires, this study interviewed U.S. Department of Veterans Affairs (VA) staff overseeing HSOs providing transitional housing under the VA's Grant and Per Diem (GPD) program to Veterans experiencing homelessness. We employed a comparative case study approach exploring GPD organizations' disaster response actions, including evacuating Veterans from wildfire‐affected areas or taking in disaster‐displaced Veterans. This article presents three themes in the GPD organizations' disaster response: (1) Organizations benefitted from close collaboration and communication with the VA during the disaster, creating a safety net to ensure Veterans' well‐being and enact rapid re‐housing to prevent homelessness; (2) Organization staff performed heroically under stressful disaster conditions; and (3) Organizations benefitted from the written disaster plans that VA requires them to create, but were not as well‐prepared for wildfires as they had been for earthquakes. As emergent threats such as the COVID‐19 pandemic, wildfires, and a very active 2020 hurricane season amplify the importance of mitigating risks, comprehensive disaster planning is needed to ensure the safety and support of people experiencing homelessness.

## 1 INTRODUCTION

In 2017 and 2018, Northern California experienced two of its deadliest and most destructive wildfire seasons in recorded history. From October 8–14, 2017, the 37,000‐acre Tubbs Fire and the 57,000‐acre Nuns Fire tore through Sonoma and Napa Counties, home to California's famous wine country. Strong winds carried the Tubbs Fire from Napa into Sonoma County before reaching parts of eastern Santa Rosa (population 176,000). Traveling at an incredible speed which left firefighters no time to issue comprehensive warnings, the Tubbs Fire jumped the US‐101 freeway, destroying 2900 homes, killing 44 people, forcing 90,000 people to evacuate, and exacerbating the demand for housing in areas already reeling from an affordable housing crisis. Unlike many wildfires whose destruction is mainly felt in sparsely populated areas on the edge of urban‐wildland areas, the Tubbs Fire wrought most of its damage within the city of Santa Rosa, causing $1.2 billion in damage and forcing a hospital to evacuate (HHS, 2019; Karklis, 2017; Rossmann, 2017; Nelson, 2017).

The following year, the 153,336‐acre 2018 Camp Fire surpassed the Tubbs Fire as the deadliest and most destructive fire in California history. It began the morning of November 8, 2018 in rural Butte County and swiftly burned through multiple residential communities, causing the bulk of its damage in the first 4 h. It continued burning through thousands of acres per day for almost 2 weeks. The town of Paradise (population 26,218) in Butte County was almost completely obliterated, as the fire destroyed 14,000 homes and killed 86 people (Baldassari, 2018; Koren, 2018; Reyes‐Velarde, 2019). The housing impacts from the Camp Fire were equally devastating, with 35,000 Paradise residents losing their housing in the blaze (Von Kaenel, 2020). Even before the fire, Butte County had already declared a homelessness crisis, with 2000 people experiencing homelessness and only a one to two percent vacancy rate (Levine, 2018). Climate experts predict that such destructive wildfires are only a precursor of the future in the Western U.S. (Laignee & Mahita, 2018).

These conditions created extreme challenges for people experiencing homelessness, disrupting both daily survival and efforts to find housing. People who are already homeless are typically the first and most severely impacted during disasters. They face disproportionate health risks due to lack of regular shelter and lack of access to information and resources to prepare for and respond to disasters (Every & Thompson, 2014; Gin et al., 2020; Kidd et al., 2020; Settembrino, 2017a, 2017b; Vickery, 2018, 2019; Walters & Gaillard, 2014; Wexler & Smith, 2015). Wildfires can present difficult recovery trajectories for people experiencing homelessness due to the enormous destruction of the existing housing stock, making it even more difficult for them to find housing, particularly in high‐cost regions (Comerio, 1998; Settembrino, 2016). Transitional housing, shelters, and other human services are often critical to ensuring their well‐being and supporting recovery (Gin et al., 2016, 2018). During the 2017–2018 wildfires, the number of evacuees seeking shelter grew almost exponentially overnight, as nonprofit homeless service organizations (HSOs) scrambled to meet the increased need from both newly displaced and preexisting homeless populations (Bay City News, 2018; Levine, 2018).

The challenge of this increased demand raises questions about the capacity of HSOs to meet the challenges posed by disasters. Beyond maintaining basic life safety on‐site, organizations take on the task of relocating residents, checking on residents' status, and expanding their outreach to people experiencing homelessness seeking services for the first time. Homeless individuals depend on this fragile safety net for basic safety and survival needs, and to support their efforts to find housing; hence, any disruption in these services can be devastating to the individuals who depend on their support to exit homelessness. Many HSOs are inadequately prepared for disasters due to challenges they face accessing preparedness resources such as technical assistance and expertise, and the constraints in staffing and funding that make it difficult to do preparedness planning (Gin et al., 2016, 2018; Vickery, 2018, 2019). HSOs often lack relationships with community partners who are essential for effective disaster response and recovery (Gin et al., 2020; Vickery, 2019). Studies of residential substance use disorder treatment programs have found that lack of specifically‐targeted guidance and funding for disaster response has hindered the ability of these programs to effectively respond to disasters (Carlisle Maxwell et al., 2009; Frank et al., 2006). Vickery, (2018, 2019) notes that the disaster precariousness of these safety net organizations often translates into gaps in the ability of people experiencing homelessness to find safe shelter during, and recover after, disasters. While there is much that we know about the vulnerability of HSOs to disasters, prior research has not examined HSO readiness for the service demands they face during disasters and what they might have done better.

### 1.1 The VA Grant and Per Diem Program

Nonprofit HSOs funded by the U.S. Department of Veterans Affairs (VA) Homeless Programs constitute a vital part of the VA's long‐term effort to end homelessness among Veterans. The VA Grant and Per Diem (VA GPD) program provides funding to non‐VA, community based HSOs to provide transitional housing and other services to Veterans experiencing homelessness. Veterans can stay up to 2 years to help them achieve permanent housing (McGuire et al., 2011; Perl, 2015; Tsai et al., 2013; VA website, 2018).

GPD grantees are inspected annually by an interdisciplinary team from the associated VA Medical Center (VAMC), led by a “GPD liaison” who monitors the Veterans' progress. The GPD inspection covers a wide scope of areas, including life safety, security, sanitation, and clinical services. In 2013, VA GPD leaders added a new regulation requiring all GPD grantees to have a written disaster plan that is coordinated with emergency managers for their local jurisdiction (VA Inspection Handbook, 2014). Prior research on the VA GPD disaster plan requirement has examined the preparedness perspectives of grantees that have not experienced a disaster (Gin, 2019b).

This study seeks to better understand, from the perspective of the VA, how these GPD organizations performed in responding to the 2017–2018 California wildfires, how prepared they were to perform these roles, and how the VA and GPD programs collaborated during the fires. As a funder, VA is unique in its adoption of a specific disaster plan requirement for its grantees. This study offers insight into how the VA, in its role as a funder, viewed their grantees' performance during an actual disaster response.

## 2 METHODS

This exploratory study sought to examine the perspectives of VA Homeless Program staff about the impacts of the 2017–2018 wildfires on GPD grantee organizations, and how they performed in responding to the safety issues and other needs that arose. The study examined VA Homeless Programs that were administering GPD grants to HSOs in Northern California, interviewing VA GPD liaisons and their supervisors staffed within VAMC Homeless Programs.

The sample of participants were purposively selected to include the two VAMC Homeless Programs and VA regional offices impacted by the 2017 Tubbs Fire. Recruitment of VA staff occurred by the research team via email and phone. At each VAMC site, one VA GPD liaison and one VA Homeless Program supervisor was interviewed. A staff member in the VA regional office, who helped coordinate the VA Homeless Program response to the wildfires, was also interviewed. A total of five respondents were interviewed. Because Northern California was also deeply affected by the 2018 Camp Fire, respondents spoke extensively about their experiences with both fire seasons.

Data were collected through qualitative, semi‐structured interviews lasting approximately 60 min. During these interviews, VA staff were asked to describe their experiences during the fires, particularly: (1) impacts to the GPD programs; (2) how the VA and the GPD programs collaborated; and (3) how well the GPD programs' disaster preparedness efforts addressed these disasters. The VA Greater Los Angeles Health Care System Institutional Review Board approved this study. Interviews were digitally audio‐recorded with the respondents' permission and transcribed.

Interview transcripts were iteratively analyzed using Atlas.ti (version 8.4.24, 2019, Berlin, Germany) qualitative analysis software. Interviews were coded using both an inductive grounded theory approach of identifying themes from the data that were most salient to VA staff perspectives as well as the three areas of deductive domains governing the interview guide (Strauss & Corbin, 1990). The first and second authors read through the transcripts and identified broad categories that were salient to understanding VA staff's perspectives. Authors were particularly interested in identifying lessons learned and ways that GPD grantees' performance would be improved in future disaster preparedness and response. Researchers sought to identify challenges, successes, impacts, and lessons from the accounts of the experiences of these nonprofit GPD programs during the wildfires. Codes were grouped according to shared findings (e.g., “I just want to commend them, because several of their staff lost their homes” and “the GPDs, they are always so great”). The resulting group codes were compared, contrasted, and sorted into themes that characterized the experiences of the VA and GPDs during the wildfires. The significance of themes was based on their substantive significance, focusing on identifying factors that either helped or hindered GPDs' performance while responding to the fires (Patton, 2014). Using a constant comparative method (Boeije, 2002), transcripts were examined to confirm that themes identified were consistent.

This study employs a comparative case study approach to explore how GPD organizations responded to Veterans' disaster needs in collaboration with VA, how these responses affected the organizations, and their Veteran participants. Comparing case studies enables an examination of similarities and differences across cases to identify factors that make a difference as well as aspects that held across the examples. Such comparisons can inform future research, thus meeting Yin's (2009) criteria that cases encompass “both discovery and theory development”, enabling the “completeness and consideration of alternate perspectives” (Yin, 2009, p. 185–188). Integrating comparative case studies with grounded theory enables us to identify common themes that run across the cases, strengthening the evidence (Halaweh et al., 2008).

## 3 RESULTS

This section presents three narratives about GPD organizations' response to the needs of Veterans during the 2017 Tubbs Fire and 2018 Camp Fire. The first and second case studies provide accounts of GPD organizations in Santa Rosa that evacuated in 2017—one evacuated to a community shelter and the second relocated Veterans to an alternate GPD location located 97 miles away in Sacramento. The third case examines the practice of GPD organizations taking Veterans, most of whom were not previously GPD participants, into their GPD programs during the 2017 and 2018 Northern California wildfires.

### 3.1 Case Study 1: A Santa Rosa Organization evacuates to a community shelter

Thousands of residents in the city of Santa Rosa were evacuated with very little advance warning in the earning morning hours of October 9, beginning at 1:30 a.m., as the Tubbs Fire jumped the freeway and spread more quickly than expected. Santa Rosa was home to two VA‐funded GPD grantees serving all‐male Veteran populations, each operated by a different nonprofit organization. The 2017 Tubbs Fire was the first and only time, as of this writing, that these GPD facilities have ever had to evacuate due to wildfires.

The first site, which has been operating GPD programs since 2010, had seven Veterans living in a four‐bedroom house with no on‐site staff, with one of the Veterans designated as a “house monitor.” On October 10, a full 24 h after community evacuations began, VA staff contacted each Veteran directly and learned that five Veterans were still staying in the house and were initially refusing to evacuate, while two had already left the area. The following day, VA staff reported mounting concerns as news and police reports indicated that the winds had turned overnight and were blowing the fires across the freeway, creating unpredictable conditions. Mandatory evacuations had been ordered in additional neighborhoods, and phone service had become unreliable. Given these concerns, VA staff and GPD program leadership agreed to order Veterans to relocate to the local evacuation shelter on October 11:

*“the wind was so strong, that there was no telling where the fire was going to jump and so…I contacted the main program and …. they wanted to evacuate the Veterans, just to be better safe than sorry. And so that was when we called the Veterans and said, ‘okay, you're going to have to go'. And they said—'no, no, we're fine'. And so the supervisor, the main supervisor at (organization) with the Grant Per Diem program. She went over to the house and physically…said—'you guys have to go'.”*

There was some confusion in the communications, as the GPD program leadership informed the VA that the Veterans had already gone to the evacuation center, while VA staff contacted the Veterans directly and learned that they were still in the house, with no intention to evacuate. When the GPD program director learned that the Veterans were reluctant to leave, she and other staff immediately traveled to the house and coordinated the evacuation. The GPD program had help from the house monitor, a former firefighter, who ultimately agreed that it would be safer to evacuate. Four of the Veterans went to the evacuation center in their own cars, while one refused to leave and remained at the house due to an alcohol dependence issue.

VA staff reported that Veterans became comfortable once they arrived at the evacuation shelter, finding a sense of community there despite their initial reluctance:

*“they really did not want to go but once they were there, they seemed to get comfortable fairly quickly…. I would say there was some camaraderie about the evacuation center.”*

The evacuated Veterans were given the option of relocating to another GPD site 25 miles away, but all opted to remain at the disaster shelter, saying that they expected to be able to safely return to the house very quickly. The first 2 days in the shelter, the house monitor helped the GPD and VA staff keep track of the Veterans for the daily evacuation status report, verifying that those evacuated were still at the shelter, and going to the house daily to check on the remaining Veteran. On the third day, the house monitor successfully found permanent housing out of the area and left the shelter. The remaining Veterans were in the shelter for 5 days. Despite the Tubbs Fire's devastating impact to the housing market, six out of the seven Veterans enrolled in the program during the fires ultimately found permanent housing before the end of their 2‐year limit in the GPD program:

*“all of the Veterans that were in the house at that time, they were able to find permanent housing…. And we were totally prepared that these Veterans would need to stay in Grant Per Diem past their time that they were supposed to have discharged. So we didn't have that problem with them, so we were very, very lucky.”*

Despite the communication and other challenges, VA staff praised the GPD staff's efforts in coordinating the evacuation and collaborating with the VA to track the Veterans daily during their stay at the shelter. They noted that the GPD staff were juggling these responsibilities during a stressful and chaotic time, with air quality problems and uncertainty about whether phone lines or freeway access would be compromised at any moment. While they were evacuating the Veterans and reporting to the VA, GPD staff were also monitoring their own evacuation situation and whether their own homes had been lost:

*“the (organization) staff actually went to the house and confirmed who was leaving the house. So that was all monitored by the (organization) staff, with constant coordination. You know, they let me know every step of the way what was going on, and I just want to commend them, because several of their staff lost their homes*.

This close collaborative relationship between the VA liaison and the GPD led to Veterans voluntarily evacuating, with monitoring from GPD staff. The next case study examines another GPD, where staff did not encounter resistance to evacuation, but had to coordinate Veterans' relocation quite closely with multiple VA liaisons.

### 3.2 Case Study 2: A Santa Rosa Organization evacuates to another gpd facility

This program, which first became a GPD grantee in 2004, had 8–10 Veterans, who evacuated for 5 days to another GPD facility 97 miles away in Sacramento that was operated by the same umbrella organization. While the Santa Rosa program was a house where Veterans lived without on‐site staff, the Sacramento facility had been operating a 42‐bed full‐service GPD program since 1995 with on‐site staff. GPD program leadership reached out to VA regional staff on October 8, when the Tubbs Fir e began, to inform them of the plan to evacuate as a precaution, due to the fire's unpredictable spread. The next day, these Veterans were transported to the Sacramento facility by the GPD staff, remaining there for 5 days until the fire had been contained. Recognizing the difficulties inherent in finding housing in an area where so much of the housing stock was destroyed by fire, the VA offered Veterans the option of enrolling in the Sacramento GPD program, but all of them were anxious to return home and ultimately opted to return to the Santa Rosa program after the evacuations were over.

The evacuation of Veterans from the Santa Rosa to Sacramento locations required some coordination with the VA because it involved Veterans being physically moved from the San Francisco VAMC catchment area to the Northern California VAMC catchment areas. Because two different VAMC GPD programs coordinated the movement of Veterans, staff at both VAMCs, as well as the VA's regional staff, had to be involved and kept informed of the evacuation. The two VAMCs' staff decided not to officially transfer the Veterans' enrollment between the GPD programs during the evacuation, as that would have also required them to transfer the per diem funding amount to the new site for that time. However, not being officially enrolled in the Sacramento GPD program meant that these Veterans were not assigned rooms and beds within that program. Instead, the GPD evacuated Veterans were all placed in a single congregate room with cots at the Sacramento GPD facility. While the VA did not need to do a comprehensive inspection of the Sacramento facility, they had to bring in staff to inspect the congregate living space to ensure that the arrangement was safe and met basic standards:

*“…So [we]had to bring out—I think it was (VA) Law Enforcement and Facilities Management to take a look at how it was set up. How the bunks were being placed, making sure we had no blockages to fire egresses, to make sure there weren't too many people in a room, and if I remember correctly, we got lucky. It was only one gender. So I didn't have to worry about female versus male safety stuff….it was all males. So once—I think when the PD and the fire people came through, we just had to….Move the cots around, just to make sure there was no blockages to any fire egresses.”*

The Sacramento VAMC staff also enrolled the Veterans in their health care system during the evacuation so their access to health care could continue uninterrupted.

VA staff overseeing the GPD programs in both regions reported that the transfer went smoothly. Once the Veterans were evacuated to Sacramento, Santa Rosa GPD program staff went to the new site daily during the evacuation to ensure their continued care and reported to the VA about the Veterans' daily evacuation status and any needs that arose. VA staff reported that, other than ensuring that the GPD program was supported and received their per diem funds, they did not have to do very much to ensure a successful disaster collaboration with the GPD. While evacuated Veterans were anxious, they were happy to be away from the danger of the fire and largely felt comfortable at the Sacramento facility, where they had access to staff support:

*“the impression I had was they were glad to be out of the area in case it caught on fire…I felt like they felt safe and they were trying to feel a part of, and I know like the case managers made sure they reached out. And that particular campus has a really nice cafeteria where all the food and stuff is made so all that stuff was made available to them. I think a couple of them kind of were anxious to get back in the area and they were… asking for answers when stuff would clear and they could go back.”*

The close relationship between GPD and VA ensured that inspections required for relocation to an alternate facility went smoothly and that Veterans were comfortable in the new location. The next case illustrates how the VA and GPD coordinated to take in disaster‐displaced Veterans who were not enrolled in GPD programs.

### 3.3 Case Study 3: Influx of veterans into GPD during wildfires

In disasters and other situations where the need arises, the VA and its GPD grantees collaborate to open vacant beds in GPD programs to any Veterans in need, regardless of whether they were previously enrolled in any VA homeless programs. During both the 2017 Tubbs Fire and the 2018 Camp Fire, Veterans living in private residences subsidized by HUD‐VASH vouchers and Veterans who presented as homeless in disaster shelters were offered vacant beds in GPD transitional housing programs, enabling them to access a plethora of supportive services designed to assist them in finding permanent housing. However, this essential role requires a significant amount of disaster coordination and communication between the VA and its GPD grantees. It requires that GPD grantees be prepared and agile to step into this role during and after disasters, even if they are not otherwise affected.

During regional disasters, this collaboration requires VA GPD program staff to work across VAMC catchment areas, obtaining bed vacancy information from their respective GPD grantees and coordinating placement with other VA Homeless programs with Veterans in need. Often, the VISN network coordinator will be involved in this coordination as well, particularly when disaster‐affected Veterans within one VAMC catchment area must be transferred to GPD programs in another VAMC's jurisdiction. VA GPD liaisons and supervisors all reported that their grantee organizations performed extremely well as VA partners when asked about their ability to absorb Veterans.

Many GPD organizations quickly stepped up to absorb these Veterans in need and did their best to ensure that displaced Veterans received needed services. As one VA liaison reported:

*“And then the GPDs, they are always so great. They always make sure the Vet has—you know, whatever…we had three Veterans who were in HUD‐VASH apartments and their apartments burned down…they spent one night in a hotel and (GPD organization) took them the next day.”*

When the 2018 Camp Fire devasted the City of Paradise in Butte County, the geographically closest GPD organizations were located 88 miles away in the Sacramento area. Sacramento GPDs were able to take in Veterans who were displaced by the Camp Fire because they were only at 90% capacity and had plenty of room. One VA GPD staff member recounted the story of a Camp Fire survivor who was experiencing homelessness, and was placed in a Sacramento‐area GPD program after being identified as a Veteran at a disaster shelter:

*“we did get one and the thing that was amazing about it…as soon as I emailed my supervisor and the HUD‐VASH Supervisor and said ‘Hey, I got this guy who decided to relocate from Paradise', they instantly gave him a HUD‐VASH voucher.”*

After the massive displacement caused by the wildfires, other VA Homeless Programs such as Supportive Services for Veteran Families and HUD‐VASH also responded quickly and proactively, expediting grants and housing vouchers to assist affected Veterans. Speaking less than a year after the fire, a GPD staff member reported that this Veteran now had access to VA health care within walking distance for the first time and was actively working toward finding permanent housing using his HUD‐VASH voucher. The GPD program was a vital resource that gave him a transitional residence, enabling him to access critical supports such as health care and a stable address to bolster his efforts to find permanent housing.

VA GPD staff also noted some challenges involved with efforts to place wildfire survivor Veterans into GPD programs. Some Veteran survivors of the 2017 Tubbs Fire sought to enter GPD programs in neighboring Marin County through this program, but VA staff were unable place them in GPD housing due to lack of capacity. VA GPD staff noted that Marin County GPD programs quickly ran out of available beds after the fires, since they were also absorbing Veterans from local disaster shelters. VA GPD staff also noted that some of the new Veterans who entered GPD programs after being displaced in the wildfires experienced challenges adapting to structured living in the GPD setting.

*“They really weren't that interested in an environment that sort of had rules and boundaries and so it's been hard to really help those Veterans…I think…these were Veterans that were just kind of living on their own in Paradise, on the edge of being homeless, but not really engaging with the community that much.”*

Undoubtedly, moving into a new area after surviving a stressful and chaotic disaster and having to adapt to a new environment with institutional rules can be a distressing experience. VA staff reported that over time, these Veterans eventually adjusted to the GPD environment and the problems were quickly resolved. In offering vacant program slots to new participants, GPD organizations play a vital role in disaster response and recovery for disaster‐affected Veterans, serving as an essential lifeline for homelessness prevention and rapid rehousing.

## 4 SUMMARY AND DISCUSSION

These case studies underscore how community‐based organizations (CBOs) providing preexisting services to marginalized populations play a crucial role in disaster response and recovery. Not only did they ensure the safety and well‐being of Veteran participants living in these VA‐funded housing programs, but they also partnered with VA to take in additional Veterans of varying degrees of homelessness status who were displaced by the wildfires. The literature notes that CBOs typically lend their vital skill sets to assist in community disaster response and recovery, often taking on additional roles, such as housing displaced individuals (Gin et al, 2018; Jenkins et al., 2015; Rivera & Nickels, 2014). These programs helped Veterans, both GPD participants and newly displaced Veterans, safely recover from a chaotic and traumatizing disaster. In the subsequent months, nonprofit organizations in the area experienced a spike in demand, driven by massive displacement. The fires' impact on an already extremely challenged housing market created a longer‐term ripple effect, complicating the efforts of transitional housing providers to help Veterans transition into permanent housing. Nonetheless, Veterans participating in these programs thrived amid the disaster because they quickly found a sense of camaraderie in disaster shelters and were able to contribute to disaster relief efforts. The role of bonding social capital in building a sense of community, both among the Veterans in the program and between the Veterans and other disaster evacuees, as often occurs in the aftermath of disasters (Jenkins, 2015; Rivera & Nickels, 2014), likely contributed to their sense of well‐being and belonging. After the fires, most were able to successfully find housing even though the fires destroyed much of the community's existing housing stock, forcing them to compete with previously housed individuals for the few units available. The support structure offered by residing in a group setting within a VA‐funded HSO likely facilitated their ability to thrive in a communal shelter setting. It may have also enabled them to sustain their progress in their housing search after the wildfires ended. Thus, community social capital among these Veterans, fostered by community organizations like GPD programs, facilitated effective disaster recovery.

Across these three case studies, the research team identified three overarching themes in how GPD organizations performed when confronted with these stressors. First, VA staff and GPD program leadership collaborated and coordinated very closely to ensure a strong safety net for Veterans, both GPD participants and new enrollees. Second, the dedication of GPD staff was evidenced in their heroic actions in diligently evacuating Veterans and tracking them during challenging disaster conditions. Finally, these experiences underscore the importance of disaster preparedness and response readiness for GPD organizations.

### 4.1 VA‐GPD collaboration and communication

Close collaboration and frequent communication between the VA and GPD program staff occurred in all three cases. During the 2017 Tubbs Fire, the VA‐GPD coordination ensured that GPD Veterans were safely moved to a community shelter, even when the Veterans initially resisted the call to evacuate, which the GPD staff did not realize. During both evacuations, both GPD and VA tracked the Veterans and communicated with them daily, ensuring that no one slipped through the cracks. While this interdependency is built into the structure of the VA GPD program, VA staff description of how the system functioned during the wildfires underscored how this relationship constitutes an additional “safety net” for ensuring Veterans' safety and well‐being.

The VA partnership also offers GPD programs the ability to transfer Veterans between catchment areas if their facilities are not able to safely continue housing them in a disaster. This close collaboration helped the second GPD move its Veterans to Sacramento. VA staff arranged for VA facilities managers to travel to the new facility to inspect sleeping areas, ensured that the GPD program would not lose any funding, and got Veterans signed up to receive health care.

GPD programs also work closely with the VA in taking in new Veterans who were unsheltered homeless or HUD‐VASH recipients displaced during disasters. This collaboration is particularly vital in helping Veterans transition from areas where there are either no GPD programs, as in Butte County, or where GPD programs were severely affected themselves, as was the case in Santa Rosa. During such situations, the VA identifies the closest GPD providers and contacts them to identify vacant beds. Being part of this vital collaborative to provide disaster‐affected Veterans with rapid rehousing resources requires GPD organizations to have highly reliable and robust preparedness plans that enable them to assess and communicate their capacity to the VA, and be agile in adapting their services to accommodate the needs of Veterans after surviving traumatic disaster experiences. Unfortunately, excess capacity was not available in all areas, as the Marin County GPDs did not have enough space to take in displaced Veterans. This suggests that if disasters are sufficiently widespread, as may be the case in climate change and pandemic conditions, the GPD network may not be able to address surge capacity concerns.

### 4.2 GPD program staff role in disaster response

VA staff noted that the GPD program staff responded heroically, going above and beyond in their handling of the chaos of moving Veterans during and after the wildfires. They “commended” the staff at the GPD organization whose Veterans who initially refused to evacuate, from the leader who personally went to the house to enforce their departure, to the staff members who checked in with Veterans daily and then reported their status to the VA. VA staff noted that GPD staff faithfully performed these tasks despite being in a state of constant uncertainty about their own homes and evacuation status. Similarly, staff at the GPD that relocated Veterans to Sacramento were praised for taking the leadership role in evacuating their residents and keeping in close communication with the VA during the process.

GPD program staff were critical in taking in non‐GPD Veterans who were displaced from regional disasters and had nowhere to go and absorbing them into their programs. One VA liaison noted: “The GPDs, they are always so great,” citing their role in preventing Veterans from becoming homeless after disasters in Northern California. GPD staff consistently provided the VA with data about their bed vacancies and took in Veterans whenever they could. VA staff noted that Veterans who were referred into Sacramento GPD programs from the 2018 Camp Fire “were kind of living on their own in Paradise, on the edge of being homeless, but not really engaging with the community that much.” Adapting to the service needs of these Veterans who may have been less familiar with navigating social service systems and were forced into GPD programs by the circumstances of the fires likely came with its own challenges, including the toll of emotional labor on GPD social service staff (Kranke et al., 2019) and the challenges likely associated with orienting these high‐need Veterans to the GPD and VA systems of care. In these stressful and demanding situations, GPD staff excelled.

### 4.3 Disaster preparedness

The high level at which GPD organizations and staff were able to perform during these stressful disaster events would not have been possible without robust disaster plans. Indeed, having disaster plans in place can improve overall preparedness, produce effective emergency operations, and mitigate the effects on people, especially among vulnerable populations (Eisenman et al., 2007; World Health Organization, 2007). The VA requires GPD organizations to have a written disaster plan, as a condition of their grants, that is coordinated with emergency managers for their local jurisdiction and “encompasses natural and manmade disasters” (VA GPD Handbook, 2014). The VA enforces compliance with this requirement through its annual inspection of GPD grantees—an inspection that also covers a wide scope of other topics, such as licensure, sanitation, clinical care, and services to Veterans. Written procedures for disaster response, especially to ensure basic life safety during disasters, are mandatory for all GPD programs.

The hallmarks of basic disaster preparedness were evident throughout the interviews where VA staff consistently praised GPD organizations for stepping up to the challenge of relocating Veterans, tracking them daily during the chaotic evacuation, and ensuring they had access to case management to ensure their well‐being and progress in exiting homelessness. Organization staff, asked to do their jobs in an anxiety‐inducing and chaotic disaster environment, may have performed well partly because their organizations had disaster plans that explicitly delineated these expectations and procedures. Similarly, the GPD organizations that took in new Veterans also displayed an agility borne from being prepared for rapidly changing contingencies. The ultimate success of the Santa Rosa GPD program that relocated to a shelter in getting all eight of its Veterans into permanent housing before their 2 years of program eligibility expired suggests that the benefits of disaster preparedness may not always be visible, but can pay dividends in the long run as program participants can be free of worry about their immediate future and focus on their long‐term objectives. The role of such safety net transitional housing programs during disasters in homeless Veterans' postdisaster success in exiting homelessness has been suggested in prior qualitative research (Gin et al., 2019a).

Although the VA requires GPD organizations to have disaster plans and procedures, VA staff indicated that GPD organizations did not internalize the need to be better prepared to respond to wildfires until after the fires. As prior research has found, the VA requirement for disaster plans and procedures did not always translate into GPD organizations becoming motivated to prepare unless prompted by actual disaster experiences, such as geographic locational risk (e.g., hurricanes) or hearing about how a similar organization struggled during a disaster (Gin et al., 2019b, p. 4). VA GPD liaisons, who oversee the inspections with guidance from VA Facilities Managers, can make suggestions during the inspection if critical elements are missing from the plan, which may prompt the GPD to improve their plans. However, because VA GPD liaisons are not required to undergo standard training in organizational disaster preparedness, their ability to identify gaps is limited to their learning from past experiences.

VA GPD liaisons and other VA staff noted that these wildfire experiences imparted valuable lessons learned for future planning. Supervisors noted that GPD organizations were not as prepared before the fires as they have learned to be now. Organizations in Northern California had always planned for earthquakes before, they said, but the 2017 Tubbs fire that destroyed residential and commercial areas in the City of Santa Rosa had shown, for the first time, that wildfires could penetrate urban communities, due to their unpredictable and rapid spread in a short amount of time. Previous wildfires had been confined to more rural, unpopulated areas in the region. One VA supervisor noted that GPD organizations should practice safety and evacuation drills regularly, especially for wildfires—a step further than the VA requirement that disaster plans include evacuation procedures, but one derived from the benefit of knowing from experience that wildfires are a real threat to their communities and that having to evacuate Veterans away from the area is a real possibility.

## 5 LESSONS LEARNED AND IMPLICATIONS

Following the 2017–2018 wildfire season, communities across Northern California have continued to struggle with a new normal of fires that challenge these communities in the late summer to early fall months annually. The question is how to ensure that Veterans living in these areas while experiencing homelessness are served by institutions that are as well‐equipped as possible. The findings of this study offer some insights.

First, the Santa Rosa evacuation case studies suggest that GPD organizations need to have very clear rules in their disaster plans about evacuation rules and protocols for Veteran clients, particularly at “scattered‐site housing,” where Veterans typically live in a group house with no program staff located on site. The lack of on‐site staff likely contributed to a sense that with the unpredictability of fast‐moving wildfire, GPDs did not want to chance conditions suddenly becoming dangerous overnight, during off‐duty hours; hence they opted to evacuate while the evacuation order was only voluntary. The GPD disaster plan may not have anticipated the veterans' resistance to evacuation. Prior disaster research has found Veterans less likely to be inclined to leave during a mandatory evacuation, possibly because of their belief that they have handled worse situations during their training or deployments (Der‐Martirosian et al., 2014). Future GPD plans should consider this contingency, and where these Veterans, living in a largely unsupervised setting, will go should evacuation be necessary. Also, the GPD that evacuated Veterans to a sister site in Sacramento was fortunate in being able to house them in a congregate room so they could remain enrolled in their home program. However, the COVID‐19 pandemic could complicate that plan in the future since Veterans could not be safely housed in a congregate facility, requiring them to be enrolled in host GPD facilities that might not have enough capacity to absorb all the evacuated Veterans. GPD disaster plans must account for these contingencies and include secondary evacuation sites in their relocation planning.

Secondly, the VA to nonprofit collaboration inherent in the GPD program structure offers a safety net for program clients and grantees, but also creates additional responsibilities for both entities. Because the VA must work to ensure that grantees continue receiving funds that are tied to bed occupancy and facilitate inspections, strong collaboration and robust coordination protocols between these two entities are essential for written disaster plans. Preparedness plans and regular drills and exercises can help GPD organizations be more agile and able to adapt quickly. Adding these to the GPD requirement would help make preparedness more meaningful.

The importance of preparedness for homeless service providers is not limited to the GPD program, as many GPD organizations also have non‐GPD programs that serve homeless clients generally, and disaster preparedness plans often permeate throughout their many programs. Disaster preparedness planning can benefit all HSOs, with modifications to address client needs. Organizations not dependent on government contracts can also be encouraged to adopt standardized disaster planning practices and requirements. National organizations (e.g., Catholic Charities, St. Vincent de Paul) can ensure that their member organizations all have a set of common disaster plan elements. All organizations can connect with peer service provider non‐profits through networks like Voluntary Organizations Active in Disasters.

The emergency management literature underscores the importance of coordinated collaborative relationships between the government and nonprofit sectors for disaster response (Kapucu, 2007). Ideally, these relationships would be well‐established during nondisaster times for maximum impact. By partnering with GPDs, this VA program exemplifies the “Whole Community Approach” by formally integrating them into a network of mutual aid that can better respond when “extreme disasters place demands on resources that can overwhelm individual responders” (Kapucu, 2007, p. 553). This close, formalized, collaboration between nonprofit GPD organizations and the VA creates the “linking social capital” that connects Veterans to institutional structures that can facilitate their disaster response and recovery, despite their own relative lack of resources (Rivera & Nickels, 2014).

Third, the 2017–2018 wildfires generated an awareness—nationally and locally—of unanticipated gaps in the required GPD disaster plans, as several VA staff noted. With the increase in human development in the wildland‐urban interface, these experiences and risks were somewhat new to these organizations (Brenkert‐Smith et al., 2006), and for these reasons, may have been somewhat unanticipated. While HSOs had comprehensive plans to address earthquake damage, they had not planned for emerging threats—such as the increased risk of wildfires in the urban‐wildland interface, infectious diseases, or climate change impacts. As novel threats, driven by climate change and an increasingly urbanized and globalized world, GPD organizations will increasingly need to prepare for and respond to emergent hazards, such as infectious diseases and pandemics such as COVID‐19. However, most HSOs are not hazard assessment experts; without external assistance, they may be unprepared for the “new normal” of emergencies and disasters they (Balbus & Crimmins, 2016), and their client population (Kidd et al., 2020) will face. Accordingly, the VA GPD program is collaborating with researchers at the Veterans Emergency Management Evaluation Center to develop technical assistance and training materials for GPD grantees to plan for these disasters.

Finally, the lessons learned from the 2017–2018 disaster experience contributed to the VA's adoption of a disaster protocol to provide all HSOs with disaster information for Veterans to follow, and to check on all Veterans participating in VA homeless programs in a disaster. It also facilitated policies to improve the VA's situational awareness about impacts to Veterans experiencing homelessness by formally integrating VA homeless program staff into the VA's emergency management roles and responsibilities (VA Homeless Programs Emergency Management Plan draft, 2019). This integration of VA GPD programs into VA's emergency management structure represented a start toward addressing a GPD grantee's complaint, articulated in prior research (Gin et al., 2019b, p. 5): “we don't have a relationship with the VA for emergency management coordination.”

This study has limitations that should be acknowledged. First, it is a multisite cross‐sectional examination of VA‐funded HSOs during a specific disaster in Northern California, and its findings may not necessarily be applicable to other disasters in different settings. Second, this study was only able to interview VA homeless program staff, so the perspectives of the GPD organizations or Veterans may yield different insights. However, the GPD Liaisons interviewed in this study were closely involved with the GPD organizations and called Veterans daily to check on their well‐being. Hence, they should be able to provide a faithful accounting of what they heard directly from GPD staff and Veterans. Future research should expand interviews to include GPD staff or Veterans. In addition, the close coordination structure between the VA and its GPD grantees may limit these findings' applicability to nonprofit organizations that lack such a deeply engaged funder to provide a back‐up form of support for clients during disasters. Finally, GPD grantees differ from other HSOs in regard to having a disaster plan mandate, further limiting applicability. We cannot assume that all HSOs will have disaster plans. However, it has been found that organizations providing housing to populations experiencing homelessness were among the best prepared and were more likely to have disaster plans than food‐provider organizations (Ritchie et al, 2010).

## 6 CONCLUSION

The experience of the 2017–2018 Northern California wildfires underscores the essential role of residential HSOs and other service providers in ensuring the safety and support of people experiencing homelessness during the chaos and confusion of disasters. Climate experts predict that the ferocity of these two fire seasons is due to become increasingly common in the Western U.S. in future years (Karklis et al., 2017; Laignee & Mahita, 2018). Indeed, at this writing, 2020 is already on track to be a more active than usual fire season in California, with enormous challenging wildfires burning in multiple sites. Organizations serving homeless individuals in the Western U.S. can expect these conditions, and the need to adapt, to persist. The lessons learned from these organizations' experiences underscores the need for HSOs to consider a broader range of hazards in their preparedness planning. Further, funding agencies should consider requiring all HSOs to have a written disaster plan to minimize the disruption of essential care to this vulnerable population. As emergent threats such as COVID‐19 and climate change effects compel homeless service providers to alter their services to reduce risk, more comprehensive disaster planning is needed to ensure the safety and support of people experiencing homelessness during disasters.

### PEER REVIEW

The peer review history for this article is available at https://publons.com/publon/10.1002/jcop.22653

## CONFLICT OF INTERESTS

The authors declare that there are no conflict of interests.

## Data Availability

The data that support the findings of this study are available on request from the corresponding author. The data are not publicly available due to privacy or ethical restrictions.

## References

[jcop22653-bib-0001] Balbus, J., Crimmins, A., et al. (2016). The Impacts of Climate Change on Human Health in the United States: A Scientific Assessment, U.S. Global Change Research Program. 10.7930/J0R49NQX

[jcop22653-bib-0002] Baldassari, E. (November 11 2018). Camp Fire death toll grows to 29, matching 1933 blaze as state's deadliest. East Bay Times. https://www.mercurynews.com/2018/11/11/crews-continue-to-battle-strong-winds-in-deadly-camp-fire/. Accessed September 20, 2020.

[jcop22653-bib-0003] Bay City News . (July 7 2018). “Number of Homeless Up in Sonoma County because of North Bay Fires” ABC7 News https://abc7news.com/society/number-of-homeless-up-in-sonoma-county-because-of-north-bay-fires/3723947/. Accessed February 24, 2020.

[jcop22653-bib-0004] Boeije, H. (2002). A purposeful approach to the constant comparative method in the analysis of qualitative interviews. Quality and Quantity, 36, 391–409.

[jcop22653-bib-0005] Brenkert-Smith, H., Champ, P. A., & Flores, N. (2006). Insights into Wildfire Mitigation Decisions among Wildland–Urban Interface Residents. Society and Natural Resources, 19, 759–768.

[jcop22653-bib-0006] Carlisle Maxwell, J., Podus, D., & Walsh, D. (2009). Lessons learned from the deadly sisters: Drug and alcohol treatment disruption, and consequences from hurricanes Katrina and Rita. Substance Use & Misuse, 44(12), 1681–1694. 10.3109/10826080902962011 19895300

[jcop22653-bib-0007] Comerio, M. C. (1998). Disaster hits home: New policy for urban housing recovery. University of California Press. https://www.ucpress.edu/book/9780520207806/disaster-hits-home

[jcop22653-bib-0008] Der‐Martirosian, C., Strine, T., Atia, M., Chu, K., Mitchell, M. N., & Dobalian, A. (2014). General household emergency preparedness: A comparison between veterans and nonveterans. Prehospital and Disaster Medicine, Apr 29(2), 134–140. 10.1017/S1049023X1400020XEpub 2014 Mar 19. PMID: 24642181; PMCID: PMC4704093. 24642181 PMC4704093

[jcop22653-bib-0009] Eisenman, D. P., Cordasco, K. M., Asch, S., Golden, J. F., & Glik, D. (2007). Disaster planning and risk communication with vulnerable communities: Lessons from Hurricane Katrina. American Journal of Public Health, 97(Suppl 1), S109–S115. 10.2105/AJPH.2005.084335 17413069 PMC1855003

[jcop22653-bib-0010] Every, D., & Thompson, K. (2014). Disaster resilience: Can the homeless afford it? Australian Journal of Emergency Management, The, 29(3), 52.

[jcop22653-bib-0011] Frank, B., Dewart, T., Schmeidler, J., & Demirjian, A. (2006). The Impact of 9/11 on New York City's Substance Abuse Treatment Programs. Journal of Addictive Diseases, 25(1), 5–14. https://doi.org/10/1300/J069v25n01_03 10.1300/J069v25n01_03 16597568

[jcop22653-bib-0012] Gin, J. L., Casey, R. J., Quarles, J. L., & Dobalian, A. (2019b). Ensuring continuity of transitional housing for homeless veterans: Promoting Disaster Resilience among the Veterans Health Administration's Grant and Per Diem Providers. Journal of Primary Care & Community Health, 10, 10. 10.1177/2150132719861262 PMC6637825 31313623

[jcop22653-bib-0013] Gin, J. L., Der‐Martirosian, C., Stanik, C., & Dobalian, A. (2019a). Roadblocks to housing after disaster: Homeless veterans' experiences after Hurricane Sandy. Natural Hazards Review, 20(3), 04019005.

[jcop22653-bib-0014] Gin, J. L., Eisner, R. K., Der‐Martirosian, C., Kranke, D., & Dobalian, A. (2018). Preparedness is a Marathon, not a Sprint: A Tiered Maturity Model for Assessing Preparedness in Homeless Residential Organizations in Los Angeles.” Natural Hazards Review, 19(1), 04017027.

[jcop22653-bib-0015] Gin, J. L., Kranke, D., Saia, R., & Dobalian, A. (2016). Disaster Preparedness in Homeless Residential Organizations in Los Angeles County: Identifying needs. Assessing Gaps. Natural Hazards Review, 17(1), 04015022.

[jcop22653-bib-0016] Gin, J. L., Levine, C. A., PhD, Canavan, D., Dobalian, A., PhD, JD, MPH (July 21 2020). Including homeless populations in disaster preparedness, planning, and response. Journal of Public Health Management and Practice, ‐ Volume Publish Ahead of Print ‐ Issue. 10.1097/PHH.0000000000001230 32701792

[jcop22653-bib-0017] Halaweh, M., Fidler, C., & McRobb, S., Integrating the Grounded Theory Method and Case Study Research Methodology Within IS Research: A Possible 'Road Map' (2008). International Conference on Information Systems 2008 Proceedings. 165. https://aisel.aisnet.org/icis2008/165/. Accessed September 24, 2020.

[jcop22653-bib-0018] Jenkins, P., Lambeth, T., Mosby, K., & Van Brown, B. (2015). Local nonprofit organizations in a post‐Katrina landscape: Help in a context of recovery. American Behavioral Scientist, 59(10), 1263–1277. 10.1177/0002764215591183

[jcop22653-bib-0019] Kapucu, N. (2007). Non‐profit response to catastrophic disasters. Disaster Prevention and Management, 16(4), 551–561. 10.1108/09653560710817039

[jcop22653-bib-0020] Karklis, L., et al. “Mapping the Wildfires in Northern California's Wine Country.” *The Washington Post*, 12 Oct. 2017. www.washingtonpost.com/graphics/2017/national/northern-california-fires/?utm_term=.65df21cc2ece

[jcop22653-bib-0021] Kidd, S. A., Greco, S., & McKenzie, K. (2020). Global climate implications for homelessness: A scoping review. Journal of Urban Health, 98, 385–393. 10.1007/s11524-020-00483-1 32965555 PMC8190258

[jcop22653-bib-0022] Koren, J. R. (December 3 2018). California plans takeover of property insurer overwhelmed by Camp Fire claims. The Los Angeles Times. https://www.latimes.com/local/lanow/la-fi-merced-insurance-paradise-20181203-story.html. Accessed September 20, 2020.

[jcop22653-bib-0023] Kranke, D., Gin, J., Der‐Martirosian, C., Weiss, E. L., & Dobalian, A. (2019). VA social work leadership and compassion fatigue during the 2017 hurricane season. Social Work in Mental Health, 18, 188–199. 10.1080/15332985.2019.1700873

[jcop22653-bib-0024] Laignee, B., & Mahita, G. (November 13 2018). “California's Wildfires Have Become Bigger, Deadlier, and More Costly. Here's Why”. Time. https://time.com/4985252/california-wildfires-fires-climate-change/. Accessed September 11, 2020.

[jcop22653-bib-0025] Levine, A. (December 3 2018). Supported by Continue reading the main story After a California Wildfire, New and Old Homeless Populations Collide. New York Times. https://www.nytimes.com/2018/12/03/us/california-fire-homeless.html. Accessed September 2, 2020.

[jcop22653-bib-0026] McGuire, J., Rosenheck, R. A., & Kasprow, W. J. (2011). Patient and program predictors of 12‐month outcomes for homeless veterans following discharge from time‐limited residential treatment. Administration and Policy in Mental Health and Mental Health Services Research, 38, 142–154. 20814735 10.1007/s10488-010-0309-9

[jcop22653-bib-0027] Nelson, L. (October 1 2017). Death toll from Northern California fires jumps to at least 34; 5,700 structures destroyed. The Los Angeles Times.

[jcop22653-bib-0050] Patton, M. Q. (2014). Qualitative research & evaluation methods. 4th, Sage Publishing. https://us.sagepub.com/en-us/nam/qualitative-research-evaluation-methods/book232962

[jcop22653-bib-0029] Perl, L. *Veterans and homelessness*. Congressional Research Service Report. RL‐34024 2015.

[jcop22653-bib-0030] Reyes‐Velarde, A. (January 11 2019). California's Camp Fire was the costliest global disaster last year, insurance report shows. The Los Angeles Times. https://www.latimes.com/local/lanow/la-me-ln-camp-fire-insured-losses-20190111-story.html. Accessed September 20, 2020.

[jcop22653-bib-0051] Ritchie, L. A., Tierney, K. J., & Gilbert, B. (2010). Disaster Preparedness among Community‐Based Organizations in the City and County of San Francisco: Serving the Most Vulnerable. Miller D. S. & Rivera J. D. Community Disaster Recovery and Resiliency: Exploring Global Opportunities and Challenges. (3–39). CRC Press.

[jcop22653-bib-0031] Rivera, J. D., & Nickels, A. E. (2014). Social capital, community resilience, and faith‐based organizations in disaster recovery: A case study of Mary Queen of Vietnam Catholic Church. Risk, Hazards & Crisis in Public Policy, 5(2), 178–221.

[jcop22653-bib-0032] Rossmann, R. (November 1 2017). Cal Fire: 4,658 homes destroyed in Tubbs fire. Santa Rosa Press Democrat. https://www.pressdemocrat.com/article/news/cal-fire-4658-homes-destroyed-in-tubbs-fire/?gallery=457C8208-A0F5-4C08-BABE-EA865EE607DB. Accessed September 11, 2020.

[jcop22653-bib-0033] Settembrino, M. R. (2016). Hurricane Sandy's impact on the Predisaster homeless and homeless shelter services in New Jersey. Journal of Emergency Management, 14(1), 7–16. 10.5055/jem.2016.0268 26963226

[jcop22653-bib-0034] Settembrino, M. R. (2017a). Exercising agency: How men experiencing homelessness employ human, social, and cultural capital to mitigate natural hazards risk. Natural Hazards Review, 18(4), 05017004.

[jcop22653-bib-0035] Settembrino, M. R. (2017b). 'Sometimes You Can't Even Sleep at Night:' Social vulnerability to disasters among men experiencing homelessness in Central Florida. International Journal of Mass Emergencies and Disasters, 35(2), 30–48.

[jcop22653-bib-0036] Strauss, A., & Corbin, J. M. (1990). Basics of qualitative research: Grounded theory procedures and techniques. Sage Publications, Inc.

[jcop22653-bib-0037] Tsai, J., Kasprow, W. J., & Rosenheck, R. A. (2013). Latent homeless risk profiles of a national sample of homeless veterans and their relation to program referral and admission patterns. American Journal of Public Health, 103, S239–S247. 24148048 10.2105/AJPH.2013.301322 PMC3969139

[jcop22653-bib-0038] U.S. Department of Health and Human Services (HHS) (November 14, 2019). The Last Stand: Evacuating a Hospital in the Middle of a Wildfire. Interview with Joshua Weil, Mitch Saruwatari, and Skip Skivington published by Technical Resources, Assistance Center, and Information Exchange, Health and Human Services Assistant Secretary for Preparedness and Response (ASPR TRACIE). https://files.asprtracie.hhs.gov/documents/aspr-tracie-the-last-stand-evacuating-a-hospital-in-the-middle-of-a-wildfire.pdf. Accessed September 11, 2020.

[jcop22653-bib-0039] U.S. Department of Veterans Affairs Grant and Per Diem Program‐ Homeless Veterans July 19, 2018. Accessed July 19, 2018, at www.va.gov/homeless/gpd.asp

[jcop22653-bib-0040] U.S. Department of Veterans Affairs Grant and Per Diem Program Inspection Handbook June 30, 2014.

[jcop22653-bib-0041] U.S. Department of Veterans Affairs Homeless Programs Emergency Management (Draft). Distributed August 2019. Accessed October 2019.

[jcop22653-bib-0042] Vickery, J. (2018). Using an intersectional approach to advance understanding of homeless persons' vulnerability to disaster. Environmental Sociology, 4, 1–12.

[jcop22653-bib-0043] Vickery, J. (2019). Homelessness and inequality in the US: Challenges for community disaster resilience. Emerging Voices in Natural Hazards Research, 145–177.

[jcop22653-bib-0044] Von Kaenel, C. (February 19 2020). Trying to Stop Long‐term Homelessness after the Camp Fire, More Than a Year Later | Homeless, in Butte County *Chico Enterprise‐Record*. https://www.chicoer.com/2020/01/02/2974642/. Accessed September 20, 2020.

[jcop22653-bib-0045] Walters, V., & Gaillard, J. C. (2014). Disaster risk at the margins: Homelessness, vulnerability and hazards. Habitat International, 44, 211–219. 10.1016/j.habitatint.2014.06.006

[jcop22653-bib-0046] Wexler, B., & Smith, M. E. (2015). Disaster response and people experiencing homelessness: Addressing challenges of a population with limited resources. J. Emergency Manage, 13(3), 195–200. 10.5055/jem.2015.0233 26150363

[jcop22653-bib-0047] World Health Organization . (2007). Risk reduction and emergency preparedness: WHO six‐year strategy for the health sector and community capacity development. https://www.who.int/hac/techguidance/preparedness/emergency_preparedness_eng.pdf

[jcop22653-bib-0048] Yin, R. K. (2009). Case study research: Design and methods (4th ed.). Library of Congress Cataloguing‐in‐Publication Data.

